# Improvement of the left atrial systolic function after a surgical reduction of the high flow arteriovenous fistula

**DOI:** 10.3389/fmed.2025.1732862

**Published:** 2026-01-09

**Authors:** Vaclav Lejsek, Anna Valerianova, Kristyna Michalickova, Kristina Buryskova Salajova, Marcela Slavikova, Marian Rybar, Jan Malik

**Affiliations:** 1 Third Department of Internal Medicine, First Faculty of Medicine, General University Hospital in Prague, Charles University, Prague, Czechia; 2 Cardiology Department, Institute for Clinical and Experimental Medicine, Prague, Czechia; 3 Department of Internal Medicine, First Medical Faculty, Charles University and Central Military Hospital Prague, Prague, Czechia; 4 Second Department of Surgery, First Faculty of Medicine, General University Hospital in Prague, Charles University, Prague, Czechia; 5 Faculty of Biomedical Engineering, Czech Technical University in Prague, Kladno, Czechia

**Keywords:** echocardiography, hemodialysis, high output heart failure, left atrial ejection fraction, left atrium

## Abstract

**Background:**

High-output heart failure (HOHF) is a distinct cardiac complication in end-stage kidney disease (ESKD) patients with high-flow arteriovenous fistulae (AVFs). While AVF flow reduction improves hemodynamics and left atrial (LA) volume, its effect on LA systolic function remains unclear.

**Objective:**

To evaluate changes in left atrial systolic function and left ventricular (LV) filling pressures following surgical AVF flow reduction in haemodialysis patients with high-flow fistulae.

**Methods:**

In this prospective, single-centre interventional study, 28 ESKD patients (mean age 63 ± 15 years) with high-flow AVFs (>1,500 mL/min) and clinical heart failure (NYHA ≥ II) underwent surgical AVF flow reduction. Echocardiographic assessments were performed before and 6 weeks after intervention. LA ejection fraction (LAEF) and LV filling pressures (E/e′ ratio) were determined from digitally stored imaging data.

**Results:**

Surgical intervention reduced AVF flow by approximately 50% [2,525 [1,388] to 1,250 [700] mL/min, *p* = 0.00006]. LA volume index decreased significantly (44.7 ± 17.3 to 38.5 ± 15.4 mL/m^2^, *p* = 0.01), accompanied by an improvement in LAEF (49.6 ± 14.9% to 53.2 ± 13.5%, *p* = 0.046). Dyspnoea improved or resolved in all patients. Baseline LAEF correlated negatively with age, LA volume index, and NYHA class, but no independent predictors of post-operative LAEF improvement were identified.

**Conclusions:**

In patients with high-flow AVF–associated HOHF, surgical AVF flow reduction leads to a significant improvement in LA systolic function alongside decreased LA volume. These findings suggest partial reversibility of atrial remodeling induced by chronic hyperkinetic circulation and highlight the potential cardiovascular benefits of AVF flow optimization in selected dialysis patients.

## Introduction

Chronic kidney disease (CKD) is a condition associated with high morbidity and mortality, affecting an estimated 700 million individuals globally, and posing a substantial economic and healthcare burden: diabetes and hypertension account for the majority of cases (1, 2).

Cardiovascular disease is the leading cause of death in the CKD population (3). In patients with end-stage kidney disease (ESKD) requiring maintenance dialysis, cardiovascular disease accounts for approximately 40% of all deaths (3). This increased cardiovascular risk is partly due to traditional atherosclerotic risk factors common in the CKD population such as hypertension, diabetes, and smoking as well as CKD-specific mechanisms, including chronic inflammation, endothelial dysfunction, accumulation of uremic toxins, and disturbances in calcium-phosphate metabolism) (4).

Chronic heart failure (CHF) is also highly prevalent in patients with CKD, affecting nearly 44% of individuals on dialysis in the United States (5). The most common phenotype is heart failure with preserved ejection fraction (HFpEF), typically associated with diastolic dysfunction due to left ventricular hypertrophy and stiffening, leading to elevated filling pressures (6, 7). A distinct mechanism contributing to heart failure in dialysis patients is hyperkinetic circulation, which may result from fluid overload, anemia, systemic inflammation, or the presence of a high-flow arteriovenous fistula (AVF) used for maintenance haemodialysis (8). Hyperkinetic circulation is a key contributor to a specific heart failure phenotype known as high-output heart failure (HOHF). This phenotype is characterized by typical signs and symptoms of heart failure in the presence of an elevated cardiac index (>3.9 L/min/m^2^) and increased circulating biomarkers, particularly brain natriuretic peptide (BNP) (9). HOHF is echocardiographically associated with a complex array of structural and functional cardiac alterations, including biventricular dilation, secondary atrioventricular valves regurgitation, left ventricular hypertrophy, increased left ventricular filling pressures, and impaired diastolic function (10, 11). Elevated left atrial (LA) pressure results in LA enlargement, and such pressure overload is a recognized risk factor for atrial fibrillation, which is also frequently encountered in the CKD population (12).

Although often considered a passive chamber by non-cardiologists, the left atrium plays a crucial role in cardiac function. Its systolic (contractile) function contributes significantly to late diastolic filling of the left ventricle, and its reservoir function helps unloading the pulmonary venous pressure, thereby mitigating pulmonary congestion. However, in ESKD patients on long-term haemodialysis, both left atrial dilation and impaired contractile function have been consistently documented (13, 14). Previous studies have demonstrated that surgical reduction of AVF flow in patients with haemodialysis-associated HOHF leads to a decrease in left atrial volume and left ventricular filling pressures. However, it remains unclear whether these changes are accompanied by an improvement in left atrial systolic function. In other words: Does LV filling pressure improvement after AVF flow reduction accompany improvement in the left atrial systolic function?” Therefore, we analyzed left atrial systolic function and left ventricular filling pressures in a cohort of patients undergoing surgical AVF flow reduction due to high-flow fistulae.

## Materials and methods

This study represents a secondary analysis of previously published prospective investigations assessing the effects of arteriovenous fistula (AVF) flow reduction on cardiovascular parameters—see references (10, 11) for details.

### Study design

This was a prospective, single-center, interventional study approved by the local institutional ethics committee. The study was approved by the Ethical committee of the General University Hospital in Prague. All participants provided written informed consent, and the study adhered to the principles outlined in the Declaration of Helsinki.

### Patient selection

Patients aged ≥18 years with a high-flow AVF (defined as AVF flow > 1,500 mL/min), documented heart failure symptoms [New York Heart Association [NYHA] class II or higher], and sinus rhythm were eligible for inclusion. Surgical AVF flow reduction was performed using one of the following techniques: banding (with or without patch) or revision using distal inflow (RUDI). Procedure selection was according to the vascular surgeon (MS) with the experience of >30 years. For this secondary analysis, we included only patients with sinus rhythm and adequate echocardiographic video loops for adequate left atrial border detections.

### Study protocol

Patients underwent clinical and imaging evaluations before and 6 weeks after the surgical intervention. Haemodialysis settings were maintained unchanged throughout the study period. To minimize the influence of volume status, all imaging examinations were conducted at a standardized time interval after the most recent dialysis session.

### Imaging and data acquisition

Echocardiographic and AVF ultrasonographic assessments were performed using a Vivid E9 system (GE Healthcare, USA), and all data were digitally stored for off-line analysis using EchoPAC software (GE Healthcare, USA).

For this secondary analysis, left atrial ejection fraction (LAEF) was derived from the archived echocardiographic video loops. The left atrial largest (=diastolic, LADV) and smallest (=systolic, LASV) volumes were measured by the Simpson method from the apical 4 and 2 chambers views. The left atrial ejection fraction (LAEF) was calculated as follows: LAEF = (LADV-LASV)/LADV and expressed in percentages. Left ventricular filling pressures were estimated using standard Doppler parameters, specifically the E/e′ ratio, where E represents the transmitral early filling velocity and e′ the early diastolic mitral annular velocity. Left ventricular diastolic function was graded according to current echocardiographic guidelines.

### Additional clinical data

Dyspnoea was assessed via NYHA functional classification. Blood pressure was measured using an automated BP monitor (Omron, Japan). All parameters were obtained non-invasively. Statistical analyses were conducted using Statistica software (StatSoft, USA).

### Statistical analyses

Distribution of all used variables was calculated. Data distribution was assessed by the Shapiro–Wilk test and visual inspection of histograms. All but one variables had normal distribution. Therefore, data are presented as mean ± SD and differences calculated using the paired *t*-test. Only Qa had a non-Gaussian distribution and is presented as median (quartile range) and differences calculated using the Wilcoxon matched pairs test. *P*-values below 0.05 were considered significant. Covariates of the left atrial ejection fraction were calculated by Pearson correlation analysis.

Power analysis: High-flow AVF is a relatively rare disease. When we were considering to perform this study, we had experience with 3 patients, in whom LAEF increased. We performed a power analysis to estimate the sample size required in the intervention group to ensure high probability of detecting the expected effect. Based on these preliminary 3 pilot patients, we assumed a mean change from baseline LAEF 51% to 56%. Using a two-tailed alpha 0.05, statistical power of 0.80 and assumed between group correlation 0.8, the analysis indicated that a minimum of 25 patients would be required. Our relatively low sample size fulfilled these assumptions.

## Results

We enrolled 28 Caucasian patients (mean age 63.3 ± 15.3 years) with high-flow arteriovenous fistulae (AVF), all undergoing maintenance haemodialysis.

The etiology of ESKD in the cohort was as follows: Hypertension (5 pts), Glomerulopathy (3 pts), IgA nephropathy (4 pts), Diabetes mellitus (5 pts), Polycystic kidney disease (3 pts), Tubulointerstitial nephritis (3 pts), Obstructive nephropathy (3 pts) and Multiple myeloma (2 pts).

### Effects of AVF flow reduction

Following surgery, AVF flow was significantly reduced by approximately 50%—from 2,525 (1,388) mL/min to 1,250 (700) mL/min, *p* = 0.00006. Dyspnoea resolved completely in 20 patients (NYHA class II prior to surgery, NYHA I after the surgery) and improved in the remaining 8 patients (from NYHA class III to II). Further hemodynamic and echocardiographic outcomes are detailed in Table 1. Two different procedures of surgical flow reduction were used: banding and RUDI. The latter was used in patients with higher initial AVF flow and led to more pronounced flow decrease. However, there was no significant difference in the heart effects (see Table 2 for details).

**Table 1:** Effects of the surgical arteriovenous fistula flow reduction.

**Variable**	**Baseline**	**6 weeks after Qa reduction (any method)**	***p*-value**
Systolic blood pressure (mmHg)	138.4 ± 19.8	134.7 ± 21.9	0.35
Diastolic blood pressure (mmHg)	81.7 ± 11.3	84.6 ±12.9	0.31
Heart rate (min^−1^)	73.5 ± 9.7	70.7 ± 10.5	0.1
Cardiac output (L/min)	**7.7** **±2.3**	**6.4** **±1.6**	**0.0016**
Left atrial volume index (ml/m^2^)	**44.7** **±17.3**	**38.5** **±15.4**	**0.01**
Left atrial ejection fraction (%)	**49.6** **±14.9**	**53.2** **±13.5**	**0.046**
E-wave (cm/s)	**0.99** **±0.27**	**0.75** **±0.27**	**< 10** ^**−6**^
Averaged e'(cm/s)	**0.09** **±0.03**	**0.08** **±0.03**	**0.02**
E/e'averaged	12.5 ± 4.7	11.2 ± 4.6	0.14

**Table 2:** Differences between surgical techniques.

**Variable**	**Banding (*n* = 14)**	**RUDI (*n* = 14)**	***P*-value of the difference between techniques**
Qa baseline (mL/min)	2,530 ± 1,288	3,419 ± 1,516	0.11
Qa change (mL/min)	**−1,180** **±1,124**	**−2,241** **±1,298**	**0.03**
CO baseline (L/min)	7.2 ±2.2	8.0 ± 2.4	0.36
CO change (L/min)	−1.0 ± 1.8	−1.6 ± 2.0	0.39
LAVi baseline (mL/m^2^)	41.9 ± 18.4	47.5 ± 16.2	0.40
LAVi change (mL/m^2^)	−2.9 ± 9.0	−9.4 ± 14.0	0.16
LAEF baseline (%)	49.9 ± 16.3	49.3 ± 13.8	0.91
LAEF change (%)	4.2 ± 11.8	3.0 ± 5.7	0.73

Qa, arteriovenous fistula flow; CO, cardiac output; LAVi, left atrial volume index; LAEF, left atrial ejection fraction.

### Correlation analyses

At baseline, left atrial ejection fraction (LAEF) showed the following significant correlations: Negative correlations with age (*r* = −0.43, *p* = 0.02), left atrial volume index (LAVi) (*r* = −0.64, *p* < 0.0001) and with NYHA functional class (*r* = −0.56, *p* = 0.02). LAEF was positively related to the diastolic blood pressure (*r* = 0.43, *p* = 0.026). No significant predictors of post-operative increase in LAEF were identified.

## Discussion

The main finding of this study is that, in patients with high-output heart failure (HOHF) caused by high-flow arteriovenous fistulae (AVFs), surgical flow reduction leads not only to a decrease in left atrial volume, but also to a significant improvement in left atrial systolic function, as reflected by the increase in left atrial ejection fraction (LAEF). Before surgical intervention, LAEF was inversely correlated with left atrial volume, patient age, and NYHA functional class, suggesting that older patients and those with more advanced heart failure tend to have more impaired atrial function.

The presence of ESKD treated by maintenance haemodialysis is associated with impaired left atrial systolic function (13–15) even among children, where it was related to fibroblast growth factor-23 levels (16). Our findings therefore suggest a unique and potentially reversible mechanism of atrial dysfunction in the context of high AVF flow. Unlike volume reduction achieved through a single haemodialysis session, which typically does not improve LAEF (14, 15), surgical AVF flow reduction appears to exert a more profound and sustained effect on left atrial function. This distinction highlights that the pathophysiological impact of chronic high-flow AVFs cannot be equated with transient fluid overload, and that chronic hyperdynamic circulation induces more complex structural and functional remodeling of the atrium. Apart from the conversion of atrial fibrillation to sinus rhythm (16), studies documented improved left atrial systolic function after physical training. In non-CKD heart failure patients, a supervised concurrent training improves the left atrial contractile function (17). Speculatively, the hyperkinetic circulation due to a high-flow arteriovenous fistula could have some similar effects as physical training.

Interestingly, the main covariates of the left atrial systolic function prior to flow reduction were age and left atrial volume also in these patients with HOHF. We observed the same associations in in ESKD patients on haemodialysis without HOHF (14). Therefore, these effects seem to be strong and independent. Indeed, left atrial systolic function worsens with age also in the general population (18). Decreased left atrial systolic function predicts development of atrial fibrillation in non-CKD heart failure patients (19).

Importantly, left atrial dysfunction should not be viewed in isolation. It frequently coexists with left ventricular diastolic or systolic dysfunction. Indeed, several studies have demonstrated a strong association between impaired LA systolic function and elevated left ventricular filling pressures (20), reinforcing the concept that left atrial performance serves as an integrated marker of overall diastolic burden.

Possible limitation of our study could be the use of the “classical” left atrial assessment by echocardiography. Nowadays, many authors prefer the left atrial strain. However, lower frame rate and other settings of the echocardiography device in this secondary analysis study prevented adequate use of the left atrial strain. Nevertheless, the left atrial systolic function assessment by strain is the least precise (21) and therefore, classical LAEF is considered as a more robust method. Other limitations include smaller sample size [caused by a relatively low occurrence of the high flow fistulas and similar to other studies (22–24)], single-center design and especially short follow-up that precludes evaluation of flow reduction durability, long-term atrial remodeling, atrial fibrillation incidence, or clinical outcomes.

Taken together, our findings support the hypothesis that reducing AVF flow in selected dialysis patients with HOHF may not only improve hemodynamics but may also reverse some aspects of atrial remodeling, potentially leading to better cardiovascular outcomes. These changes are similar to that after a physical training in heart failure patients. Further prospective studies with larger cohorts and longer follow-up are warranted to assess the clinical significance of these echocardiographic improvements, particularly with respect to atrial arrhythmias, exercise tolerance, and quality of life.

## Data Availability

The raw data supporting the conclusions of this article will be made available by the authors, without undue reservation.
