# Correction: Improvement of the left atrial systolic function after a surgical reduction of the high flow arteriovenous fistula

**DOI:** 10.3389/fmed.2026.1784889

**Published:** 2026-02-13

**Authors:** Vaclav Lejsek, Anna Valerianova, Kristyna Michalickova, Kristina Buryskova Salajova, Marcela Slavikova, Marian Rybar, Jan Malik

**Affiliations:** 1 Third Department of Internal Medicine, First Faculty of Medicine, General University Hospital in Prague, Charles University, Prague, Czechia; 2 Cardiology Department, Institute for Clinical and Experimental Medicine, Prague, Czechia; 3 Department of Internal Medicine, First Medical Faculty, Charles University and Central Military Hospital Prague, Prague, Czechia; 4 Second Department of Surgery, First Faculty of Medicine, General University Hospital in Prague, Charles University, Prague, Czechia; 5 Faculty of Biomedical Engineering, Czech Technical University in Prague, Kladno, Czechia

**Keywords:** echocardiography, hemodialysis, high output heart failure, left atrial ejection fraction, left atrium

Author Marian Rybar was erroneously spelled as Martin Rybar.

The original version of this article has been updated.

